# Heat treatment dependent cytotoxicity of silicalite-1 films deposited on Ti-6Al-4V alloy evaluated by bone-derived cells

**DOI:** 10.1038/s41598-020-66228-x

**Published:** 2020-06-11

**Authors:** Ivana Nemcakova, Ivan Jirka, Martina Doubkova, Lucie Bacakova

**Affiliations:** 1 Institute of Physiology of the Czech Academy of Sciences, v.v.i., Videnska 1083, 142 20 Prague 4, Czech Republic; 2 J. Heyrovsky Institute of Physical Chemistry of the Czech Academy of Sciences, v.v.i., Dolejskova 3, 182 23 Prague 8, Czech Republic; 3 Second Faculty of Medicine, Charles University, V Uvalu 84, 150 06 Prague 5, Czech Republic

**Keywords:** Chemistry, Cell biology, Biomaterials - cells, Implants, Synthesis and processing

## Abstract

A silicalite-1 film (*SF*) deposited on Ti-6Al-4V alloy was investigated in this study as a promising coating for metallic implants. Two forms of *SFs* were prepared: as-synthesized *SFs* (*SF-RT*), and *SFs* heated up to 500 °C (*SF-500*) to remove the excess of template species from the *SF* surface. The *SFs* were characterized in detail by X-ray photoelectron spectroscopy (XPS), by Fourier transform infrared spectroscopy (FTIR), by scanning electron microscopy (SEM) and water contact angle measurements (WCA). Two types of bone-derived cells (hFOB 1.19 non-tumor fetal osteoblast cell line and U-2 OS osteosarcoma cell line) were used for a biocompatibility assessment. The initial adhesion of hFOB 1.19 cells, evaluated by cell numbers and cell spreading area, was better supported by *SF-500* than by *SF-RT*. While no increase in cell membrane damage, in ROS generation and in TNF-alpha secretion of bone-derived cells grown on both *SFs* was found, gamma H2AX staining revealed an elevated DNA damage response of U-2 OS cells grown on heat-treated samples (*SF-500)*. This study also discusses differences between osteosarcoma cell lines and non-tumor osteoblastic cells, stressing the importance of choosing the right cell type model.

## Introduction

The ageing population and the increasing search for a longer, more active and healthier life, has led to an increasing demand for better biomedical implants^1^. Orthopedic implants designed to replace, regenerate or support bones affected by a degenerative disease or by an injury have to withstand frequent high loads. It is therefore highly desirable for material density values, mechanical strength values, and Young’s elastic modulus values to be as close as possible to those of the bone itself, in order to prevent future loosening of the implant^2–4^. High biocompatibility of implant materials with the tissue and with the body fluids is also required for good integration and healing without undesirable effects^5^.

Because of their superior mechanical properties, metallic alloys are the most widely-used materials for the fabrication of load-bearing orthopedic implants^3,4^. However, when they are exposed to load and to corrosive biological environments, these materials release metallic ions, which can cause allergic reactions, inflammation, pain and bone resorption^4^. Alarmingly, the accumulation of metallic ions caused by the wear of implant surfaces is considered to be the main reason for implant failure^6–8^. This release of metallic ions is significantly reduced in Ti-6Al-4 V implants, due to the inert TiO_2_ layer that spontaneously forms on the surface of the alloy. Although this layer has a higher rate of spontaneous recovery after mechanical disruption than other metallic materials (e.g. Co-Cr-Mo, Zr-Nb alloys and 316L stainless steel^9^), Ti-6Al-4 V implants may still release potentially harmful vanadium and aluminum ions into the host body^10,11^. A number of studies have reported a cytotoxic and genotoxic effect of vanadium^12,13^, while aluminum has been associated with the induction of neurotoxicity and neurodegenerative diseases^14^. Moreover, it has been claimed that exposure to aluminum has a negative effect on the structural properties of rat bones, and also reduces bone mineralization resulting in bone loss^15,16^.

Various types of functional coatings (such as hydroxyapatite-based and carbon-based nanodiamond, DLC or fullerene films) are currently under investigation for minimizing the release of metallic ions and wear particles, and also for enhancing the osseointegration and the durability of metallic implants^17–21^. Among others, MFI zeolites (such as aluminosilicate ZSM-5 and pure-silica silicalite-1) are very attractive materials for coating implants, due to their high thermal and chemical stability^22^ together with their high wear and corrosion resistance^23–25^. Another important advantage is the low elastic modulus of MFI films, which can match the modulus of the host cortical bone (about 30 GPa, while the modulus of bare Ti-6Al-4 V alloy is about 110 GPa^24,26^). Minimizing the difference between the modulus of the implant and the modulus of the host bone should reduce stress-shielding, resulting in better osseointegration and prolonged durability of the implant *in vivo*^27^.

In addition, MFI coatings are generally considered to have good biocompatibility and osteoconductivity, as has been reported by Bedi *et al*. and by Yong *et al*.^25,28,29^. However, investigations of MFI zeolites have mostly been performed on MFI aluminosilicate (ZSM-5), which has potentially harmful aluminum ions in its structure. In order to completely eliminate the possibility of aluminum ion release from orthopedic implants into the host body over time, an aluminum-free coating, such as pure-silica MFI (silicalite-1), should be used. Our previous studies have shown good biocompatibility of a silicalite-1 film (***SF***) deposited on an Si(100) wafer and on stainless steel substrates^30–32^. Since the parameters of ***SFs*** (e.g. the wettability, porosity, charge and topology of the layers) can be modified during synthetic and post-synthetic procedures, various modifications to silicalite-1 layers have been investigated. As-synthesized coatings have improved the adhesion and proliferation of human osteosarcoma cell lines Saos-2 and MG-63. Interestingly, calcined films (at 500 °C) displayed lower cell numbers and an increased DNA damage response of osteosarcoma cell lines^30,33^. However, a similar increase in DNA damage was also observed in cells grown on the reference stainless steel without ***SFs***.

We therefore decided to investigate this matter more deeply in the present study. Ti-6Al-4 V alloy, widely used in clinical applications, was chosen as a reference alloy instead of stainless steel. An immortalized cell line obtained from non-tumor human fetal osteoblasts (hFOB 1.19), better representing the normal osteoblast genotype and phenotype^34,35^, was used instead of previously used malignant osteosarcoma cell lines, e.g. MG-63 and Saos-2. This study investigates not only the positive behavior of the cells, e.g. adhesion, proliferation and metabolic activity, but also potential membrane and DNA damage as well as oxidative stress and immune activation of cells grown on ***SFs***. Human osteosarcoma cell line U-2 OS, with intact TP53 and RB tumor-suppressor genes, was used for an evaluation of the potential genotoxicity and immunogenicity of the silicalite-1 coatings, because it was more prone than hFOB 1.19 cells to DNA damage and to TNF-alpha secretion (based on our preliminary data).

## Results and Discussion

**Figure 1:**
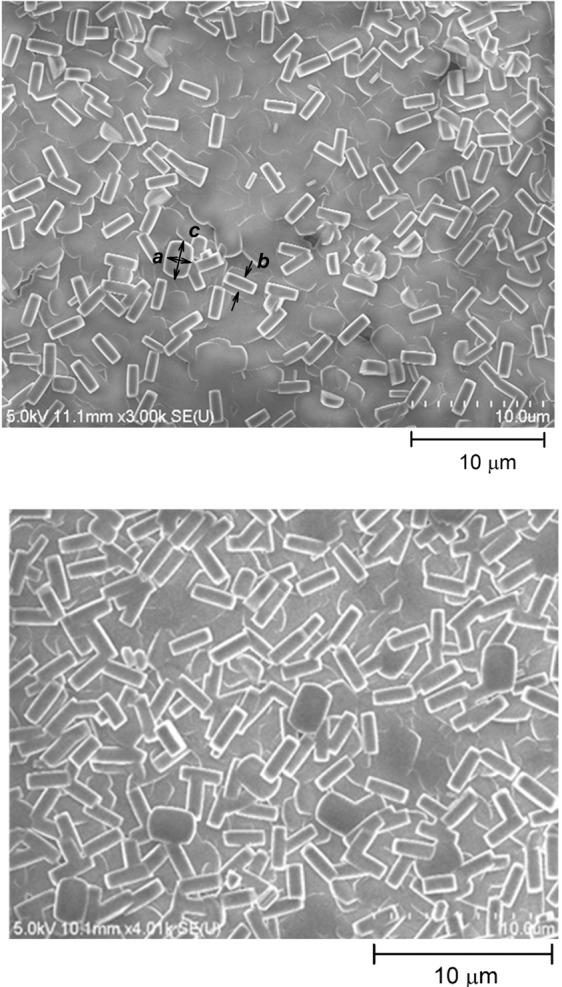
SEM image of the as-synthesized silicalite-1 film (***SF-RT***; above) and the silicalite-1 film heated up to 500 °C (***SF-500***; below). The surface of the films is covered by crystals with dimensions a ~1.9 μm, b ~0.9 μm, c ~2.8 μm.

Typical SEM images of ***SF-RT*** and ***SF-500*** are depicted in Fig. 1. The heating rate and the calcination temperature that were used were found to be the maximum levels for treating ***SFs*** without inducing any cracks. No morphological changes were induced by calcination of ***SFs***, so the morphology of both samples (***SF-RT*** and ***SF-500***) is comparable. The both zeolite films were composed of a compact ***b***-oriented layer *I* covered by a discontinuous layer *II* of ***a***, ***b*** oriented crystals (Fig. 1). The overall surface morphology of the ***SFs*** is therefore the superposition of the surface irregularities of layers *I* and *II*. ***SFs*** with an analogous morphology were characterized in our previous paper by atomic force microscopy (AFM)^31^. The roughness of the ***b***-oriented crystals of layer *I* was 11–21 nm (RMS). Layer *II*, which covered ~50% of the surface of layer *I*, induced surface irregularities with a typical height of ~600 nm. The ***SF*** surface thus exhibits a hierarchical morphology with a combined nano- and submicro- (often also called meso-) roughness.

**Figure 2:**
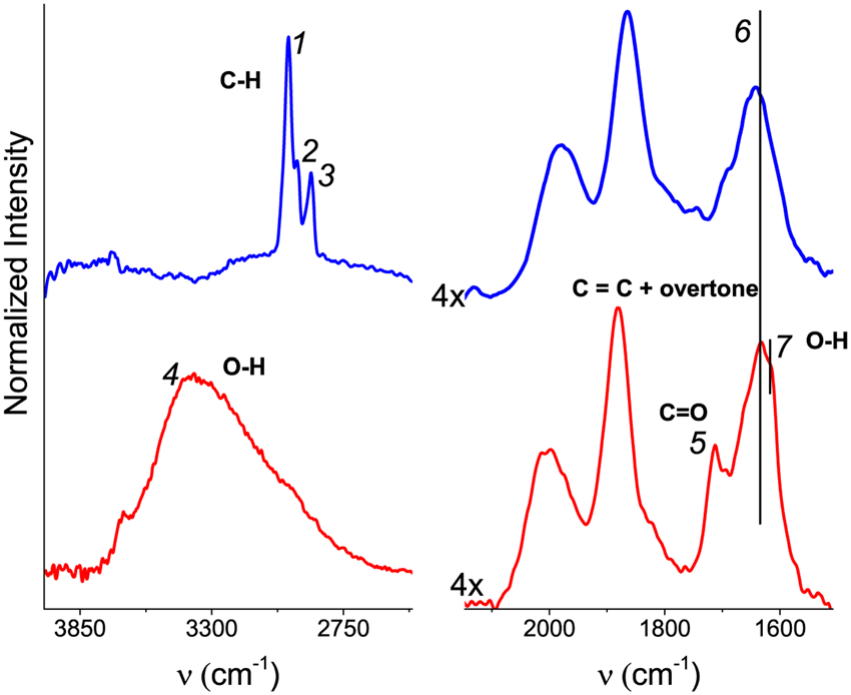
FTIR spectra of the as-synthesized silicalite-1 films (***SF-RT***; above) and the silicalite-1 films heated up to 500 °C (***SF-500***; below).

A comparison of the FTIR and XPS methods shows that heat treatment induced a highly heterogeneous distribution of carbonaceous residua. The presence of TPAOH in ***SF-RT*** is reflected in the FTIR spectrum by bands ***1***, ***2*** and ***3*** at ~2980 cm^−1^, ~2943 cm^−1^ and 2881 cm^−1^ (Fig. 2, upper spectrum^36^;). In the spectrum of ***SF-500*** (Fig. 2, bottom spectrum), these bands were replaced by another intensive broad band ***4*** at ~3394 cm^−1^ (physisorbed water), which provided evidence of desorption of template species from the bulk of ***SF-500***. According to FTIR, the porous structure of ***SF-500*** (i.e. its inner surface) was therefore free of template species. However, some hydrocarbon residua remained in ***SF-500***. Line ***5*** at 1712 cm^−1^, assigned to cyclohexanone^33^, appeared in the spectrum of this sample. The intensity of overtone band ***6*** of ***SF-500*** at ~1642 cm^−1^ was slightly increased due to an overlap with another band of aromatic species. The shoulder ***7*** at ~1617 cm^−1^ of ***SF-500*** belongs to physisorbed water^33^.

XPS reflects the chemical composition of the sample within the information depth (~10 nm). According to XPS, the surface region of sample ***SF-RT*** was enriched by carbonaceous phase. While the value of *c(N)* (0.06) was close to *c(N)*^*s*^ (0.04), the value of *c(C)* (0.81) was substantially higher than the stoichiometric value (0.50, cf. Table 1). Absence of the N 1s line in the photoelectron spectrum of ***SF-500*** (*c(N)* = 0.00) showed complete degradation of TPAOH by calcination. The concentration of the carbonaceous phase decreased in this sample, but some carbonaceous phase remained in the outer surface region of ***SF-500***.

**Table 1:** Estimated values of c(C), c(N), c(O) of samples ***SF-RT*** (the as-synthesized silicalite-1 film) and ***SF-500*** (the silicalite-1 film heated up to 500 °C) in comparison with their stoichiometric values and calculated values of d (nm).

	*c(C)*	*c(N)*	*c(O)*	*d* (nm)
***SF-RT***	0.81	0.06	1.85	0.71
***SF-500***	0.48	0.00	1.48	3.18
*c*^*s*^*(x)*	0.50	0.04	2.00	

The *c(O)* values of ***SF-RT*** and ***SF-500*** are lower than the stoichiometric values *c(O)*^*s*^ (Table 1). These lower values can be explained as a consequence of attenuation of the emitted photoelectrons by the carbonaceous overlayer on the sample. This attenuation is stronger for the O 1s line than for the Si 2p line, due to sufficiently different values of the relevant inelastic mean free paths of photoelectrons *λ*. The thickness *d* (nm) of the carbonaceous overlayer can be estimated as follows:

1 $${\rm{c}}({\rm{O}})={\rm{c}}{({\rm{O}})}^{{\rm{s}}}.\exp (\,-\,{\rm{d}}/{\lambda }^{O1{\rm{s}}}).\exp ({\rm{d}}/{\lambda }^{{\rm{Si}}2{\rm{p}}})$$

where *c(O)*^*s*^ is the stoichiometric concentrations of oxygen in silicalite, *λ*^*O1s*^ and *λ*^*Si2p*^ are the inelastic mean free paths of the photoelectrons emitted from the O 1s and Si 2p levels^37^. The values of *d* calculated from equation (1) demonstrate substantial enrichment of the surface of ***SF-5*00** by the carbonaceous layer (cf. Table 1).

**Figure 3:**
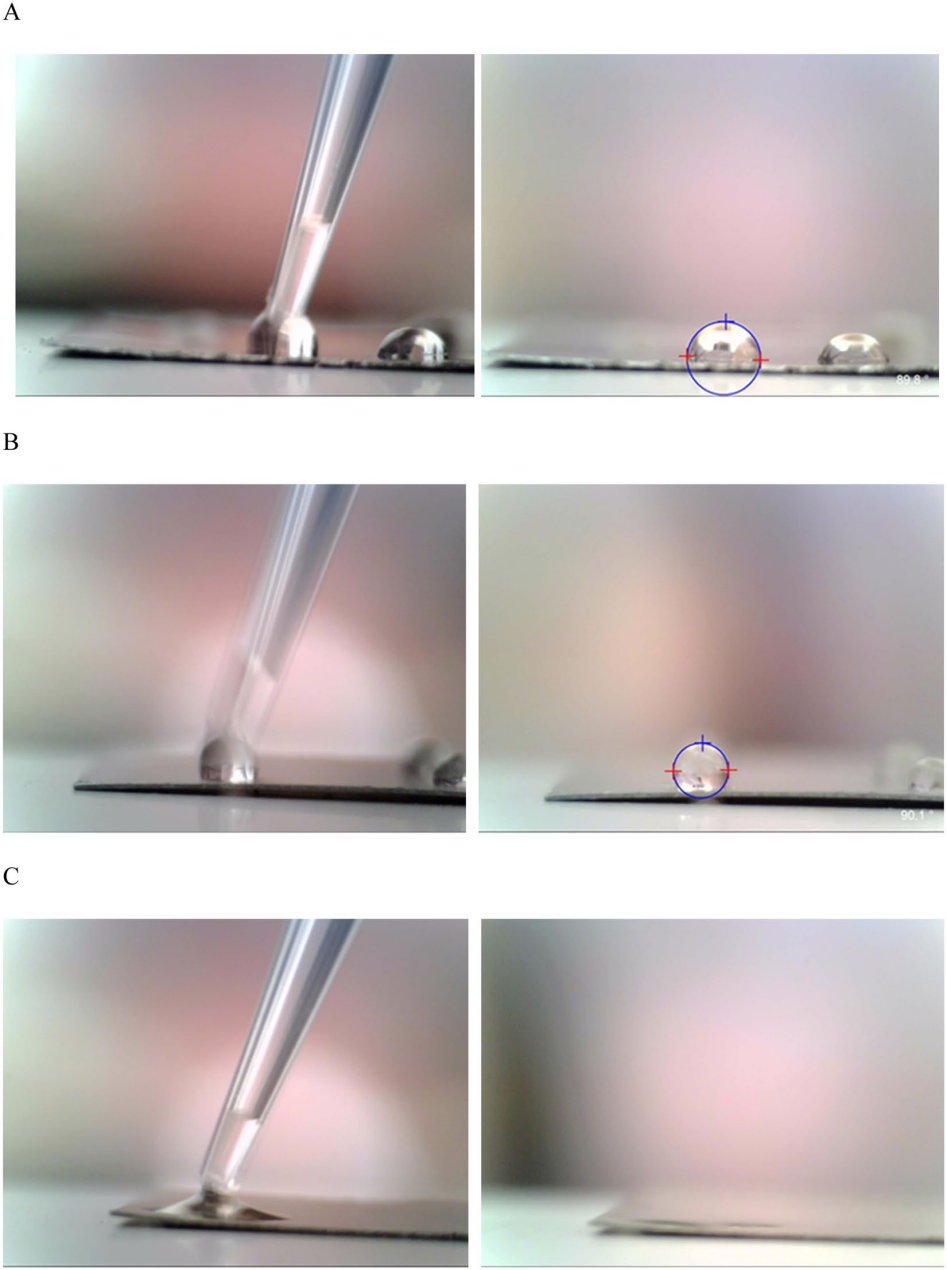
Images of the water droplets just attached (left) and 30s after they had been deposited (right) on the uncoated surface of Ti-6Al-4V (**A**) and on the as-synthesized silicalite-1 film (***SF-RT***; (**B**)). The water droplet just attached (left) and 1s after deposition (right) on ***SF-500*** (**C**).

The images of water drops on the surfaces of the Ti-6Al-4V substrate and sample ***SF-RT*** and ***SF-500*** are summarized in Fig. 3. The contact angle values are summarized in Table 2. The contact angle estimated for ***SF-RT*** is in good agreement with the previously published value of ***SFs*** on the Si(100) substrate^31^. The relatively high value of WCA observed for ***SF-RT*** is caused by the enrichment of hydrophobic C-containing species on the outer surface of the sample, as observed by XPS (Table 1). After the water drop was deposited on the surface of sample ***SF-500***, it was rapidly adsorbed into the bulk of the zeolite due to liberation of the porous structure of this sample (in line with the FTIR results). The water contact angle for ***SF-500*** therefore could not be properly estimated.

**Table 2:** The mean static water drop contact angle values measured on the uncoated surface of Ti-6Al-4V and on the as-synthesized silicalite-1 film (***SF-RT***). + depicts a significant difference from Ti-6Al-4V with p ≤ 0.05.

Sample	WCA (deg)	Standard deviation
Ti-6Al-4V	72	6.3
***SF***-***RT***	90 ^**+**^	3.5

**Figure 4:**
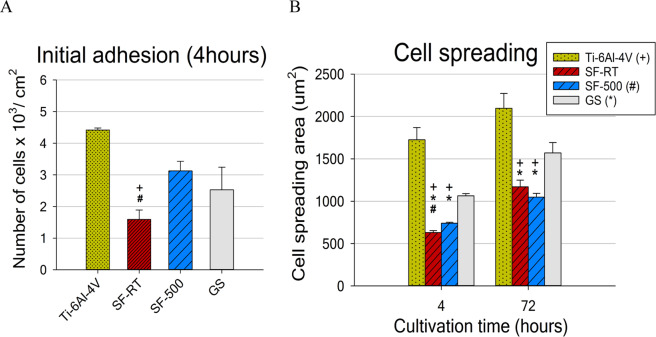
Number of hFOB 1.19 cells adhered 4 hours after seeding (**A**) and their spreading area after 4 hours of cultivation and after 72 hours of cultivation (**B**) on bare Ti-6Al-4V samples (Ti-6Al-4V), on samples coated with as-synthesized silicalite-1 films (***SF-RT***) or with silicalite-1 films heated up to 500 °C (***SF-500***), and on the control microscopic glass coverslips (GS). + depicts a significant difference from Ti-6Al-4V, *Difference from GS and # difference from ***SF-500*** with p ≤ 0.05.

The results for initial adhesion showed that calcined ***SF-500*** supported the initial adhesion of hFOB 1.19 cells better than ***SF-RT***. The cell number values **(**Fig. 4A**)** and also the spreading area of the cells 4 hours after seeding **(**Fig. 4B**)** were higher for ***SF-500*** than for ***SF-RT***. Moreover, the number of initially adherent cells (4 hours after seeding) on ***SF-500*** was comparable to the cell numbers found on the reference alloy and also on the control microscopic glass coverslips (GS; Fig. 4A**)**. However, the initial cell spreading area (4 hours after seeding) was better on the reference alloy and on the control GS than on both evaluated ***SF*** materials **(**Fig. 4B). The cells on ***SF-500*** and on ***SF-RT*** had a more rounded morphology than the cells with the polygonal spindle-shaped morphology found on Ti-6Al-4V and GS. Although the spreading of the cells grown on the ***SF*** samples improved over time, the differences between the spreading of the cells grown on the ***SF*s** and on the two control materials were still apparent after 3 days of cultivation. The difference in cell spreading area between ***SF-500*** and ***SF-RT*** disappeared over time (no differences were found on day 3; Fig. 4B).

In summary, the initial adhesion of hFOB 1.19 cells, evaluated by cell numbers and cell spreading area, was better supported by ***SF-500*** than by ***SF-RT***. Unlike the number of cells grown on ***SF-RT***, the number of cells grown on ***SF-500*** in the first 24 hours (4 hours and 24 hours after seeding) was comparable to the cell number found on the reference GS and Ti-6Al-4V. These findings differ from our earlier results observed on human osteosarcoma cell line (Saos-2), which preferred ***SF-RT*** and ***SF-300*** over ***SF-500***^30^. While no decrease in the number of Saos-2 cells grown on all ***SF*** samples was found, the spreading of cells on all ***SF*** samples was slightly but significantly lower than on the reference GS and on stainless steel. Similarly, another study of ours, performed on a different osteosarcoma cell line (MG-63), showed a markedly lower spreading area without a lower density of cells grown on ***SF-RT*** than on the reference GS and on reference Si(100) substrates^31^. The lower spreading of both osteosarcoma cell lines grown on ***SF*** samples is in accordance with the lower spreading of the non-tumor osteoblastic cell line in the present study. This effect can be attributed mainly to the high hydrophobicity of the ***SF-RT*** samples (water contact angle around 90°; Table 2), which is significantly higher than the hydrophobicity of the reference Ti-6Al-4V (around 72°; Table 2) and GS (around 60°^21^). When the average water contact angle of ***SF-RT*** was found to be around 90° or higher, the spreading of the cells was markedly more impaired than when the contact angle was below 85°^30,31^. Similarly, heat treatment of ***SF*** samples has been shown to increase the surface wettability of ***SFs*** (Table 2 and^30^), which could explain why ***SF-500*** supported the initial adhesion of hFOB 1.19 cells better than ***SF-RT***.

As shown in Fig. 1, and from our previous publications, ***SFs*** consist of two layers of **b**-oriented and **a**, **b**-oriented crystals forming a combination of the nano-scale and the submicro-scale surface morphology^31^. Nano-structured surfaces in particular, and often also submicro-structured surfaces, are generally preferred by osteoblasts, because they mimic the *in vivo* environment of the extracellular matrix and its components, including hydroxyapatite crystals and collagen fibrils^38^. Surface structures much smaller than the cell itself (e.g. nano-scaled and submicro-scaled) are generally more favorable to integrin-mediated osteoblast functions (e.g. cell adhesion, spreading and proliferation)^39,40^. However, a number of studies have described the negative effect of submicro-scale and the micro-scale roughness on cell adhesion, morphology, cytoskeleton development and cell proliferation (for a review see^39,41^). For example, a study by Wu *et al*. observed that hFOB 1.19 cells proliferated more slowly on titanium surfaces with submicro-roughness than on titanium surfaces with micro-roughness^42^. Moreover, the proliferation of cells grown on the submicro-structured titanium surface was markedly slower than the proliferation of cells grown on the reference culture plate, which is in agreement with our results. Thus, not only the high hydrophobicity but also the high roughness of the ***SF*** surface could cause lower adhesion of hFOB 1.19 cells grown on both ***SF*** samples. Similarly, studies by Kohal *et al*. and Setzer *et al*. have shown that hFOB 1.19 cells grown on rougher titanium or zirconia surfaces adhered in lower numbers, exhibited a rounded cell morphology with a poorly developed cytoskeleton and slower proliferation than cells grown on smoother surfaces and on smooth culture flasks^43,44^. However, these studies also showed that the rougher surfaces promoted a higher expression of osteogenic markers *in vitro* and quicker healing, with greater biomechanical strength of the bone–implant integration *in vivo*. Likewise, a study comparing a flat zirconia surface with a hierarchically-structured surface (i.e., a combination of nano-, micro-, meso- morphology) did not show any improvement in the adhesion or in the proliferation of osteoblasts; however, the expression of osteogenic markers was higher in cells grown on the surface with the hierarchical morphology. Moreover, the hierarchically-organized roughness also promoted better osseointegration and greater biomechanical strength *in vivo*^45^. Thus, slower cell adhesion and proliferation observed *in vitro* do not necessary mean poor and slow osseointegration *in vivo*.

**Figure 5:**
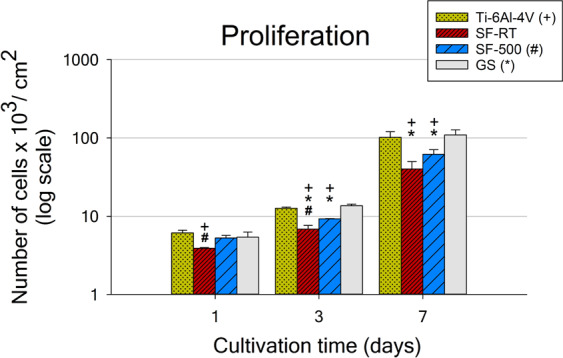
Numbers of hFOB 1.19 cells grown for 1, 3 and 7 days on bare Ti-6Al-4V samples (Ti-6Al-4V), on samples coated with as-synthesized silicalite-1 films (***SF-RT***) or with silicalite-1 films heated up to 500 °C (***SF-500***), and on the control microscopic glass coverslips (GS). + depicts a significant difference from Ti-6Al-4V, *difference from GS and # difference from ***SF-500*** with p ≤ 0.05.

In compliance with the data obtained for initial adhesion, hFOB 1.19 cells reached higher cell numbers when grown on ***SF-500*** than when grown on ***SF-RT*** also on day 1 and 3 **(**Fig. 5**)**. A similar trend was observed on day 7, but the differences were not proven to be significant. While the numbers of cells grown on ***SF-500*** were comparable to the numbers found on both reference materials for day 0 (4 hours after seeding) and for day 1, lower cell densities were observed on ***SF-500*** on days 3 and 7 (Fig. 5). The initial adhesion and the later cell number data show that the cells grown on both ***SF*** samples proliferated more slowly, with longer doubling times in comparison with the cells grown on the reference materials. The doubling times in the exponential phase of cell growth (occurring between days 1 and 3) for both investigated ***SF*** samples were 57.5 hours, while the cells grown on the reference Ti-6Al-4V needed only 44.7 hours **(**Table 3**)**. All these values were significantly higher than the doubling time of cells grown on the reference GS (36.2 hours), matching the doubling time of the hFOB 1.19 cells stated by the cell supplier (36 hours; ATCC).

**Table 3:** Doubling times of hFOB 1.19 cells in the exponential growth phase, calculated from the cell numbers obtained on day 1 and on day 3.

DT D1-3	Hours
Ti-6Al-4V	44.7 ± 3.1*
***SF-RT***	57.5 ± 7.5*
***SF-500***	57.5 ± 4.2*
GS	36.2 ± 3.7

*Depicts a significant difference from GS, p ≤ 0.05.

**Figure 6:**
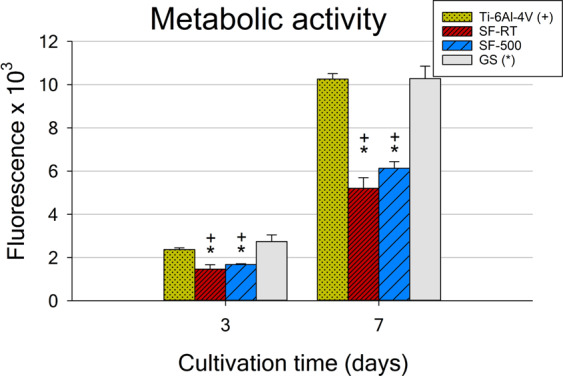
Metabolic activity of hFOB 1.19 cells grown for 3 and 7 days on bare Ti-6Al-4V samples (Ti-6Al-4V), on samples coated with as-synthesized silicalite-1 films (***SF-RT***) or with silicalite-1 films heated up to 500 °C (***SF-500***), and on the control microscopic glass coverslips (GS). + depicts a significant difference from Ti-6Al-4V and *shows the difference from GS with p ≤ 0.05.

The results for the metabolic activity of hFOB 1.19 cells corresponded with the data obtained from the cell numbers, with lower metabolic activity of cells grown for 3 and 7 days on ***SF-RT*** and ***SF-500*** than on both reference materials (GS and Ti-6Al-4V alloy; Fig. 6). Although the metabolic activity of cells grown on ***SF-500*** was slightly higher than the metabolic activity of cells on ***SF-RT*** for both evaluated time intervals, the differences were not proven to be statistically significant.

The cell numbers and the metabolic activity of hFOB 1.19 cells show that the cells grown on both ***SF*** samples proliferated significantly more slowly than the cells grown on the reference GS and Ti-6Al-4V, without any significant difference between ***SF-RT*** and ***SF-500*** (Table 3). The slower proliferation of hFOB 1.19 cells on the ***SF*** samples is in disagreement with our previous studies, where the osteosarcoma cell lines (Saos-2 and MG-63) grown on ***SF-RT*** and ***SF-300*** deposited on different substrates, i.e. Si(100) or stainless steel, showed a comparable proliferation rate or even a higher proliferation rate than the cells grown on the reference controls^30–32^). These differences are probably caused by differences between the osteosarcoma cell lines and the non-tumor osteoblast cell line. Although osteosarcoma cell lines (such as Saos-2, MG-63, U-2 OS etc.) are widely used as an osteoblastic model due to their convenient fast and unrestricted cell division, they differ from normal osteoblasts at various levels.

Osteosarcoma cell lines are derived from malignant bone tumors. They possess a variety of chromosomal abnormalities and gene mutations, resulting in inactivation of tumor-suppressor genes (mainly TP53 and RB) or other components of their regulation pathways, and in overexpression of various oncogenes (such as C-FOS, C-JUN, C-MYC etc.)^46–48^. While Rb protein is a major regulator of cell cycle progression and cell cycle arrest (i.e., cell growth inhibition and indirectly induced apoptosis), p53 protein is responsible not only for cell cycle arrest but also for the DNA damage response (DNA repair) and for inducing apoptosis (cell death)^49,50^. Osteosarcoma cell lines with mutations or deletions of these genes fail to enter cell cycle arrest or apoptosis and continue with rapid and unrestricted cell division, lacking physiological features such as contact inhibition. These cell lines often proliferate independently from external signals from their environment, and are therefore resistant to chemotherapy and radiation^51,52^. Moreover, a study by Lin *et al*. reported that, unlike hFOB 1.19 cells, a high percentage of MG-63 cells were able to resist anoikis and apoptosis when they were prevented from adhering to a culture dish for 7 days. The survival of MG-63 cells was attributed to their ability to form large compact multicellular aggregates^53^.

However, primary osteoblasts and non-tumor osteoblast cell line hFOB 1.19 are derived from normal (non-pathological) tissues, and they therefore have the normal expression of tumor suppressors and oncogenes. Studies comparing osteosarcomas with hFOB 1.19 cells showed downregulated expression of microRNAs and proteins acting as tumor suppressors in osteosarcomas but not in hFOB 1.19 cells or in normal adjacent tissues (NATs)^48,54–59^. Similarly, other microRNAs and proteins acting as oncogenes were found to be overexpressed in osteosarcomas but not in hFOB 1.19 cells or in NATs^54,58,60^. Thus, unlike osteosarcomas, normal osteoblastic cells have a carefully regulated progression through the cell cycle and are highly dependent on external signals from their immediate environment (e.g. growth factors, hormones and cytokines expressed by neighboring cells and stimuli from the quality of cell adhesion to the extracellular matrix or to the substrate)^61^. Studies comparing primary osteoblasts and hFOB 1.19 cells with osteosarcoma cell lines showed differences in cell proliferation and death, and also in the production of many specific proteins important in cell adhesion, proliferation, cell cycle regulation, cell signalling, telomere lengthening, apoptosis and other cellular processes^62–65^. Moreover, a publication comparing various osteosarcoma cell lines with human primary fibroblasts (a cell type very close to osteoblasts, which are sometimes called “sophisticated fibroblasts”^66^), revealed chromosomal instability, extensive aneuploidy and high occurrence of atypical cell division of osteosarcoma cells. For example, in MG-63 cells (which are p53 deficient), 15% of centrosome aberrations and 24% of atypical cell division was found, while Saos-2 cells (which are both p53 deficient and Rb deficient) exhibited even higher values (25% and 40%, respectively). In contrast, the U-2 OS osteosarcoma cell line, which has both p53 and Rb intact, showed a low occurrence of centrosome aberrations (3%) and atypical cell division (2%) with similar values to primary human fibroblasts with 3% and 0% of these phenomena, respectively^67^. Similarly, a karyotype analysis of hFOB 1.19 cells showed only minimal chromosomal abnormalities in the range typical for primary cells^35^.

All these differences between osteosarcomas and non-tumor osteoblastic cells also result in different cell behavior in response to the same artificial culture substrate or in response to the same culture conditions. Zhang *et al*. showed that interleukin 6 treatment promoted proliferation, migration, invasion, clonogenicity and chemoresistance of both MG-63 cells and U-2 OS cells. However, the same treatment did not increase any of these features in hFOB 1.19 cells^68^. Another example of a different response of MG-63 cells and hFOB 1.19 cells to the same treatment has been observed by Liu *et al*., revealing markedly higher sensitivity of hFOB 1.19 cells to melatonin resulting in changes in the proliferation rate^69,70^. Similar results were found in studies evaluating the biocompatibility of various potential biomaterials. Kraus *et al*. showed that hFOB 1.19 cells were more susceptible to the cytotoxicity of various dental resin monomers than MG-63 cells and Saos-2 cells^71^. Likewise, hFOB 1.19 cells were reported to be more sensitive than Saos-2 cells to PHPE polymer (poly(4-hydroxy-L-proline ester)) cytotoxicity^72^. A study comparing various chitosan scaffolds showed the highest activity of alkaline phosphatase (a marker of osteogenic differentiation) in hFOB 1.19 cells grown on a chitosan/glucan/hydroxyapatite scaffold, while Saos-2 cells preferred a scaffold composed of chitosan/glucan/hydroxyapatite with tricalcium phosphate granules^73^. Moreover, Wilkesmann *et al*. demonstrated cytotoxicity of 45S5-bioactive glass for U-2 OS, HOS and Saos-2 osteosarcomas but not for MG-63 osteosarcoma, osteoblasts and bone marrow stromal cells^74^. A different response of various osteosarcomas and osteoblasts was also found during stimulation by an electromagnetic field, by hydrostatic pressure or by mechanical stretching^75–77^.

**Figure 7:**
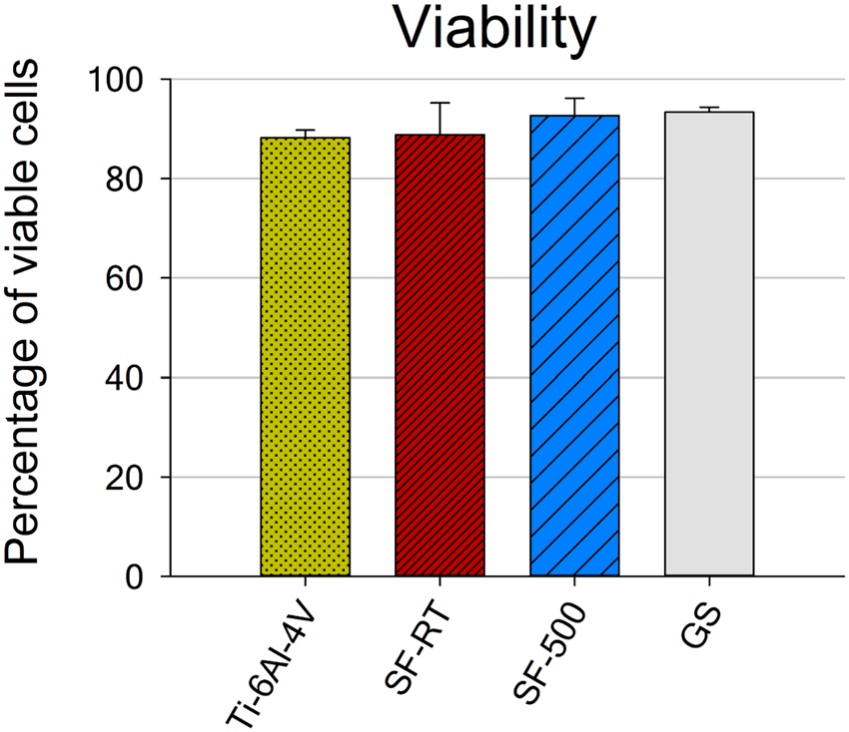
Viability of hFOB 1.19 cells grown for 7 days on bare Ti-6Al-4V samples (Ti-6Al-4V), on samples coated with as-synthesized silicalite-1 films (***SF-RT***) or with silicalite-1 films heated up to 500 °C (***SF-500***), and on the control microscopic glass coverslips (GS). The percentage of viable cells (y-axis) means the percentage of cells which were negative for trypan blue staining and therefore had intact cell membranes.

In order to observe the reason behind the slower proliferation of hFOB 1.19 cells grown on the ***SF*** samples, potential cell membrane damage and the generation of reactive oxygen species (ROS) were investigated. Trypan blue dye, used for the cell viability assessment, penetrates only through the damaged cell membrane and stains non-viable cells. According to the results, both ***SF*** samples showed lower cell numbers, metabolic activity and cell spreading, but the viability of hFOB 1.19 cells grown on these layers was not impaired. Both ***SF*** samples promoted high viability of hFOB 1.19 cells (around 90%; i.e. 90% of the cells were negative for trypan blue staining), which was comparable to the viability of cells grown on the reference Ti-6Al-4V and on the reference glass coverslips (Fig. 7). No increase in membrane damage of cells grown on ***SFs*** for 7 days was found. Similar results were observed in our previous study, where deposition of ***SF-RT*** on an Si(100) substrate did not impair the viability of cultured MG-63 osteoblast-like cells. The cell viability even improved in comparison with the viability of cells grown on bare Si(100)^31^.

**Figure 8:**
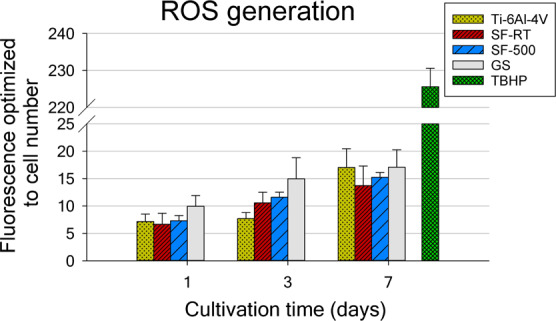
Generation of reactive oxygen species by hFOB 1.19 cells grown for 1, 3 and 7 days on bare Ti-6Al-4V samples (Ti-6Al-4V), on samples coated with as-synthesized silicalite-1 films (***SF-RT***) or with silicalite-1 films heated up to 500 °C (***SF-500***), and on the control microscopic glass coverslips (GS). The measured florescence values are optimized to the cell number.

The formation of intracellular ROS by hFOB 1.19 cells grown on the ***SFs*** samples was estimated with the use of the commercially available DCFDA kit. The raw fluorescence data corresponded with the trend of the cell numbers data, with lower values for cells grown on ***SF-RT*** and on ***SF-500*** than on both reference materials (GS and Ti-6Al-4V). The values optimized per cell number revealed no increased ROS formation by cells grown on the ***SF*** samples (Fig. 8). The ROS formation data for both ***SFs*** were comparable with the values for the reference GS and Ti-6Al-4V.

In addition, the potential immunogenicity and genotoxicity of ***SFs*** were investigated. Human U-2 OS cells were used to assess the immune activation and the DNA damage response to ***SFs***, because this cell line has both p53 and Rb intact, and it was also more prone to DNA damage and to TNF-alpha secretion than hFOB 1.19 cells (based on our preliminary data).

**Figure 9:**
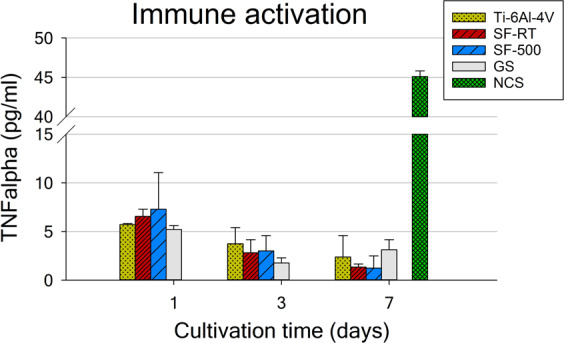
TNF-alpha secretion by U-2 OS cells grown for 1, 3 and 7 days on bare Ti-6Al-4V samples (Ti-6Al-4V), on samples coated with as-synthesized silicalite-1 films (***SF-RT***) or with silicalite-1 films heated up to 500 °C (***SF-500***), and on the control microscopic glass coverslips (GS). NCS – neocarzinostatin treatment (700 ng/mL for 1 hour), a positive control.

On the one hand, the results for immunogenicity showed no increase in TNF-alpha secretion into the culture medium by U-2 OS cells grown on ***SF*** samples for 1, 3 and 7 days (Fig. 9). The TNF-alpha secretion data for both ***SFs*** were comparable to the values on the reference GS and Ti-6Al-4V. Similar results with no increase in TNF-alpha secretion were found for hFOB 1.19 cells (data not shown).

**Figure 10:**
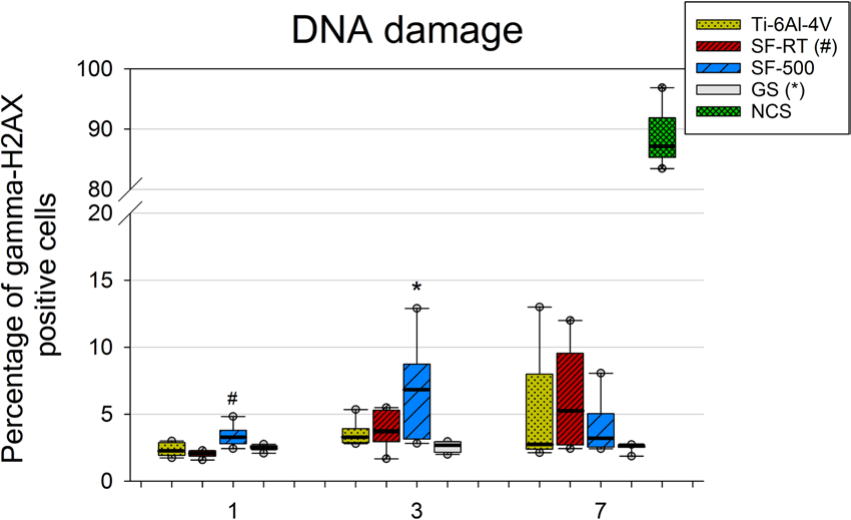
Flow cytometry of the marker of DNA double-strand breaks gamma-H2AX (phosphorylated histone H2AX) in U-2 OS cells grown for 1, 3 and 7 days on bare Ti-6Al-4V samples (Ti-6Al-4V), on samples coated with as-synthesized silicalite-1 films (***SF-RT***) or with silicalite-1 films heated up to 500 °C (***SF-500***), and on the control microscopic glass coverslips (GS). The box plot bold black central line shows the median, and its outer edges represent the 1^st^ and 3^rd^ quartile. *Indicates a significant difference from GS, and # shows the difference from ***SF-RT*** with p ≤ 0.05.

On the other hand, a double-strand DNA damage analysis of the U-2 OS cells revealed an increased DNA damage response of U-2 OS cells grown on ***SF-500*** for 1 day and for 3 days (Fig. 10). The amount of gamma-H2AX (phosphorylated histone H2AX) on day 1 was slightly but significantly higher in cells grown on ***SF-500*** than on ***SF-RT***; however, the amount did not differ significantly from the amount found in the cells grown on the reference GS and Ti-6Al-4V. Similarly, the percentage of cells with double-strand DNA breaks, cultured for 3 days, was higher in cells grown on ***SF-500*** than in cells grown on the reference GS. No significant differences among the samples were found on day 7, due to a higher data spread.

These results are in agreement with our previous study, which evaluated the photodynamic and genotoxic properties of ***SFs*** deposited on stainless steel^33^. In full agreement with our results, this paper showed increased induction of double-strand breaks in U-2 OS cells grown on ***SF-500***, but not on ***SF-RT*** (labelled as ***SF-AS***). This study also revealed the presence of residual polycyclic aromatic hydrocarbon species (PAHs) created during the heat treatment of ***SF-500***. A series of various PAH molecules, including naphthalene, fluorene, phenanthrene, anthracene, fluoranthene and pyrene, were identified and quantified on the ***SF-500*** surface^33^. The cytotoxicity and the genotoxicity of these molecules, including the genotoxicity mechanism, have been rigorously discussed in our previous study and also in studies of other authors^33,78–81^. Since the formation of intracellular ROS was not elevated in the cells grown on the ***SF-500*** sample, oxidative stress is probably not responsible for the observed DNA damage. Thus, the creation of DNA double-strand breaks found in our study could be attributed to the direct binding of PAH molecules or their metabolites to DNA. PAH metabolites have previously been described to bind to the N3 and N7 positions of guanine and adenine in the DNA molecule, creating depurinating DNA adducts^82,83^. These DNA adducts can inhibit the activity of DNA polymerase, resulting in an accumulation of DNA damage^84^. Moreover, the loss of modified DNA bases creates apurinic sites, which can cause permanent mutations or double-strand breaks in DNA^85^. Increased DNA damage in the presence of PAH molecules also explains the previously observed lower cell densities and lower spreading areas with a poorly developed cytoskeleton of Saos-2 cells grown on ***SF-500*** deposited on stainless steel^30^.

In summary, the poor adhesion and proliferation of hFOB 1.19 cells grown on ***SF-RT*** samples can be attributed to the high hydrophobicity of ***SF-RT*** (around 90°). Heat treatment of ***SFs*** improved the initial adhesion of hFOB 1.19 cells due to the increased wettability of ***SF-500***. However, heat treatment did not improve the proliferation of hFOB 1.19 cells, as would be expected from their enhanced initial adhesion. An investigation of the reason behind the slower proliferation of hFOB 1.19 cells grown on both ***SFs*** showed no increased membrane damage or elevated ROS generation of hFOB 1.19 cells grown on ***SFs***. Moreover, no increased immune activation of hFOB 1.19 or U-2 OS cells was found. However, heat treatment of ***SFs***, previously shown to induce the formation of PAH molecules on the surface of ***SF-500***, caused the increased formation of double-strand breaks in U-2 OS cells grown on ***SF-500***, resulting in the slower proliferation of the cells grown on these samples. Thus, the preparation of ***SFs*** with an open microporous structure (created by the process of calcination at 500 °C^30^), and at the same time with a non-toxic outer surface, proved to be a challenging issue. Our future work will therefore be focused on further optimizing of the cleaning procedures of the outer surface of post-synthesized silicalite-1 coatings.

## Materials and Methods

### Preparation of silicalite-1 films (*SFs*)

Polished titanium-aluminum-vanadium foil (Ti-6Al-4V, thickness 0.1 mm, purchased from Goodfellow Metals (GB)), was cut into 1 × 1 cm coupons. The edges of the coupons were rubbed down to eliminate contamination introduced by cutting the foil, and were then washed using an ultrasonic bath in sequence for 10 minutes (150 W) in acetone and isopropyl alcohol, after which they were rinsed in ethanol and were dried in air. The ***SFs*** were synthesized *in situ* from the reaction mixture of tetrapropylammonium hydroxide (TPAOH), tetraethylorthosilicalite (both Sigma-Aldrich) and deionized water, as described in the literature^86^. The reaction mixture was aged for 2 hours. The synthesis proceeded for 3 hours in a Teflon-lined autoclave under autogenous pressure at 165 °C. The coupons were oriented upside down during synthesis. The as-synthesized ***SFs***, assigned as ***SF-RT***, were sonicated in deionized water (150 W, 10 minutes).

One part of the ***SFs*** was heat treated as follows: heating rate 1 °C min^−1^ up to 500 °C, at which level the samples were maintained for 4 hours, after which they were cooled at a rate of 1 °C min^−1^ in a stream of dry air (300 mL min^−1^). The calcination temperature was chosen to minimize the presence of volatile condensed aromatic species, which had been observed in our preceding study^33^. The heat-treated samples were designated as ***SF-500***.

### Characterization of *SFs*

The morphology of the ***SFs*** was characterized by Scanning Electron Microscopy (SEM) and their chemical composition was characterized by Fourier Transfer Infrared Spectroscopy (FTIR) and by X-ray Photoelectron Spectroscopy (XPS). While FTIR is a bulk sensitive method, the information depth of XPS is limited by the inelastic scattering of the emitted photoelectrons, and does not exceed ~10 nm.

#### SEM

The morphology of the ***SF*** samples was characterized using an S 4800-I scanning electron microscope (Hitachi). Acceleration voltage of 1 keV was applied.

#### XPS

An Omicron Nanotechnology ESCAProbe P spectrometer (Omicron Nanotechnology GmbH, DE) was used to measure the photoelectron spectra. XPS analysis was performed at a pressure of ~10^-8 ^Pa. The X-ray source was monochromatic at 1486.6 eV. The photoelectron spectra were measured at low resolution (survey spectra in the energy region 0–1000 eV with a step size of 0.6 eV) and at high resolution (C 1s, N 1s and Si 2p spectra in 30 eV scans with step size 0.1 eV). The concentrations of C and O atoms, assigned as *c(C)* and *c(O)*, were evaluated as C/Si and O/Si atomic ratios calculated from the integral intensities of the C 1s, O 1s and Si 2p photoelectron spectra normalized on the probability of photoemission^87^.

#### FTIR

The infrared spectra were recorded using an FTIR Nicolet 6700 spectrometer under an ambient atmosphere in reflection mode. The spectra were normalized relative to the unit height of the most intense skeletal vibration.

#### Water Contact Angle (WCA) measurements

The wettability of the Ti-6Al-4V before and after coating with ***SFs*** was characterized by WCA measurements. Static water drop contact angles were estimated using the SEE system (Masaryk University, Brno, Czech Republic). The water drop that was used was 0.5 μL in volume; the contact angle was measured at 30 seconds after it was deposited on the surface using an Eppendorf pipette (Eppendorf, Hamburg, Germany). Ten drops were used in each experiment.

### Cells and culture conditions

Two types of silicalite-1 films deposited on the reference Ti-6Al-4V alloy were investigated in this study – as-synthesized films (***SF-RT)***, and films after calcination at 500 °C (***SF-500***). Ti-6Al-4V alloy without silicalite-1 films (labelled as Ti-6Al-4V) and a microscopic glass coverslip (GS) were used as reference materials. All samples were sterilized in an autoclave (Tuttnauer Co. Ltd., Israel) and were inserted into polystyrene 24-well culture plates (well diameter 15.4 mm; TPP Techno Plastic Products AG, Switzerland).

An immortalized cell line obtained from non-tumor human fetal osteoblasts (hFOB 1.19; Cat. No. CRL-11372; ATCC, USA) was used for an investigation of cell adhesion, proliferation, metabolic activity and viability. The cells were seeded at an initial density of 6,500 cells/cm^2^ and were grown on the investigated samples for 4 hours, 1 day, 3 days and 7 days in a 1:1 mixture of Ham’s F12 medium and Dulbecco’s Modified Eagle’s medium without phenol red (Gibco, Fisher Scientific, USA) supplemented with 10% fetal bovine serum (FBS; Gibco, Fisher Scientific, USA) and geneticin G418 (0.3 mg/mL; Gibco, Fisher Scientific, USA) at a temperature of 33.5 °C in a humidified air atmosphere containing 5% CO_2_.

Human osteosarcoma cell line U-2 OS (Cat. No. HTB-96; ATCC, USA) was used for an evaluation of the immune activation and the DNA damage response in densities ranging from 4,000 through 16,300 to 54,300 cells/cm^2^ for 7 day, 3 day and 1 day long cultivation, respectively. U-2 OS cells were then cultured in Dulbecco’s Modified Eagle’s medium (Gibco, Fisher Scientific, USA) supplemented with 10% FBS and gentamicin (40 μg/mL; Sandoz, Novartis, Switzerland) at 37 °C in a humidified air atmosphere containing 5% CO_2_ for 1, 3 and 7 days.

### Evaluation of cell adhesion, proliferation, doubling time

The hFOB 1.19 cells grown on all samples were rinsed in phosphate-buffered saline (PBS, Sigma-Aldrich, USA) and were fixed with 4% paraformaldehyde for 15 minutes in analyzed time intervals of 4 hours, 1 day and 3 days after seeding. The fixed samples were permeabilized with 0.1% Triton X-100-Tween in PBS (Sigma-Aldrich, USA) and were stained with a solution of Texas Red C_2_-maleimide (20 ng/mL; Molecular Probes, Invitrogen, USA) and Hoechst #333258 (5 μg/mL; Sigma-Aldrich, USA) in PBS for 1 hour in dark at room temperature (RT). A minimum of 10 microphotographs of random areas were taken for each sample (a minimum of 30 images in total for each experimental group) by an epifluorescence microscope (Olympus IX-71 with a DP71 digital camera, Olympus Corp., Japan).

The spreading areas and the cell numbers were evaluated from microphotographs that were taken with the use of Atlas image analysis software (Tescan Ltd., Czech Republic). The cell numbers were expressed as cell population densities per cm^2^, and the data from days 1 and 3 (the exponential cell growth phase) were used for calculating the cell population doubling times according to the following equation:

$$DT=log2\frac{t-{t}_{0}}{log{N}_{t}-log{N}_{{t}_{0}}}$$

Time intervals after seeding are represented by *t*_*0*_ (earlier) and *t* (later), whereas *Nt*_*0*_ and *Nt* represent the number of cells at particular time intervals.

The cell numbers and the cell viability on day 7 were assessed automatically by a Vi-CELL XR analyzer (Beckman Coulter, USA). The samples were transferred to fresh 24-well culture plates, were rinsed in PBS and were detached by Trypsin-EDTA solution (Sigma-Aldrich, USA), which was subsequently neutralized by adding a fresh culture medium. The concentration of the cell suspension was then measured by a Vi-CELL XR analyzer.

### Evaluation of the metabolic activity of the cells

A resazurin assay based on the activity of mitochondrial dehydrogenases was used to investigate the metabolic activity of hFOB 1.19 cells. All evaluated samples were transferred to fresh 24-well culture plates with fresh PBS after 3 and 7 days of cultivation. The samples were then incubated in a mixture of a fresh culture medium with 10% FBS and resazurin at a final concentration of 40 mM (Sigma–Aldrich, USA) at 33.5 °C in a humidified air atmosphere containing 5% CO_2_. The fluorescence of the culture medium-resazurin mixture was measured (Ex/Em = 530/590 nm) by a Synergy HT Multi-Mode Microplate reader (BioTek, USA) after 4 hours of incubation. A solvent mixture without cells was used as a blank control.

### Evaluation of potential membrane damage (cell viability) and ROS generation (oxidative stress)

The potential cell membrane damage was detected by trypan blue staining, which was performed during cell counting in the Vi-CELL XR analyzer on day 7 of cultivation. Trypan blue dye is a membrane impermeable molecule, so only the cells with damaged cell membranes (i.e. dead or dying cells) are positive for trypan blue staining. For each experimental group, 50 images from three parallel samples were evaluated within one experiment.

The generation of intracellular reactive oxygen species (ROS) by hFOB 1.19 cells was assessed by a DCFDA kit (2′,7′ –dichlorofluorescin diacetate; Abcam, Cambridge, UK) according to the manufacturer’s protocol. Briefly, all evaluated samples with adhered cells were transferred to fresh 24-well culture plates with fresh PBS after 1, 3 and 7 days of cultivation. The samples were then stained with DCFDA (10 µM for 25 minutes) at a temperature of  33.5 °C. The samples were subsequently rinsed with PBS and were cultured in a fresh culture medium for another 2.5 hours. The fluorescence of 2′,7′ –dichlorofluorescein (DCF; formed by ROS activity) was measured (Ex/Em = 485/535 nm) by a Synergy HT Multi-Mode Microplate reader (BioTek, USA). Stained material samples without the cells were used as a blank control. hFOB 1.19 cells treated with tert-butyl hydrogen peroxide (TBHP; 250 µM) for 2.5 hours (right after DCFDA staining) were used as a positive control for ROS generation.

### Evaluation of immune activation and the DNA damage response

Human osteoblast-like cell line U-2 OS was used instead of hFOB 1.19 cells for these analyses. Unlike other osteoblast-like cell lines, the U-2 OS cell line has both p53 and Rb genes working, which is needed for the successful DNA damage response of the cells. This cell line was also more prone to TNF-alpha secretion and DNA damage response after neocarzinostatin treatment than the hFOB 1.19 cells (based on our preliminary data).

The potential immune activation of U-2 OS cells was evaluated by the amount of TNF-alpha molecules secreted into the culture medium by cells grown on the investigated samples. Cell culture media were collected from the samples after 1, 3 and 7 days of cultivation with U-2 OS cells. The media were subsequently centrifuged at 2,000 g for 10 minutes to remove the cell debris. The concentration of TNF-alpha in the culture supernatants was assessed by a commercial human TNF-alpha ELISA kit (ab46087; Abcam, UK). The absorbance of the mixture was measured at 450 nm by a Synergy HT Multi-Mode Microplate reader (BioTek, USA). A solvent mixture without cells was used as a blank control, while a solvent mixture obtained from U-2 OS cells treated with neocarzinostatin (700 ng/mL incubated for 1 hour 3 hours before the culture media were collected; Sigma-Aldrich, USA) was used as a positive control of TNF alpha secretion.

To evaluate the DNA damage response of U-2 OS cells grown on ***SF*** samples, Gamma-HA2X antibody (specific to phosphorylated histone H2AX) assessed by flow cytometry was used as a marker of DNA double-strand breaks. All evaluated samples were transferred to fresh 24-well culture plates with fresh PBS after 1, 3 and 7 days of cultivation. Cells were then detached from the samples by Trypsin-EDTA solution (Sigma-Aldrich, USA), which was subsequently neutralized by adding a fresh culture medium. Cells were centrifuged at 300 g for 5 minutes to remove the culture medium and were then rinsed again in PBS and were fixed in cold 70% ethanol (Lach-Ner, Czech Republic) at −20 °C. Fixed cells were centrifuged at 700 g for 5 minutes, were rinsed in cold PSB with 5% FBS, and were incubated with Alexa Fluor 488 anti-H2AX-Phosphorylated (Ser139) antibody (5 µg per 1 million cells; clone 2F3; Bio-Legend, USA). After 1 hour of incubation, the cells were rinsed again in cold PSB with 5% FBS and were subsequently resuspended in PBS. The stained cells were analyzed using an Accuri C6 Flow Cytometer (BD Biosciences, USA). U-2 OS cells treated with neocarzinostatin (700 ng/mL for 1 hour; Sigma-Aldrich, USA), fixed 2 hours after the treatment, were used as a positive control for the DNA damage response.

### Statistical analysis

From three to seven samples were evaluated for each experimental group and time interval, including the reference controls. At least 10 microphotographs of randomly selected areas were taken for each sample (a minimum of 30 images in total for each experimental group and time interval) for cell numbers and spreading areas. The size of the cell spreading area was measured in a minimum of 100 or 250 individual cells for each experimental sample group on day 0 (4 hours) or on day 3, respectively.

The results are presented as mean ± S.E.M. (Standard Error of Mean) or as median with quartiles, maximum and minimum values and outliers in box plots. All charts were made using SigmaPlot 13.0 (Systat Software Inc., USA). A statistical analysis of the acquired data was performed using SigmaStat 4.0 (Systat Software Inc., USA). Multiple comparison procedures were carried out by the one-way ANOVA, Student–Newman–Keuls test or by Kruskal–Wallis one-way ANOVA on Ranks, followed by Dunn’s multiple comparison test. Values of p < 0.05 were considered statistically significant for all experiments.

## Conclusions

Although silicalite-1 has previously been shown by widely-used osteosarcoma cell lines to have good biocompatibility, the present study has revealed impaired adhesion and slower proliferation of human non-tumor osteoblastic cells (hFOB 1.19) grown on both ***SF*** samples. Heat treatment of ***SFs*** improved the initial adhesion of hFOB 1.19 cells, but did not improve their proliferation. An investigation of the potential cytotoxicity of ***SFs*** did not reveal increased membrane damage, elevated ROS generation or increased immune activation of hFOB 1.19 or U-2 OS cells grown on both ***SF*** samples. Importantly, an evaluation of the potential genotoxicity of ***SF*** samples showed increased induction of double-strand breaks in U-2 OS cells grown on ***SF-500***, but not on ***SF*** without heat treatment (***SF-RT)***. Special care should be therefore given to choosing the right post-synthesis treatments of silicalite-1, and also to the use of the right osteoblast model for an *in vitro* evaluation.
